# Oral health and plaque microbial profile in juvenile idiopathic arthritis

**DOI:** 10.1186/s12969-019-0387-5

**Published:** 2019-12-16

**Authors:** Sriharsha Grevich, Peggy Lee, Brian Leroux, Sarah Ringold, Richard Darveau, Gretchen Henstorf, Joel Berg, Amy Kim, Elizabeth Velan, Joseph Kelly, Camille Baltuck, Anne Reeves, Hannah Leahey, Kyle Hager, Mitchell Brittnacher, Hillary Hayden, Samuel Miller, Jeffrey McLean, Anne Stevens

**Affiliations:** 1 Seattle Children’s Hospital, 4800 Sand Point Way NE, Seattle, WA 98105 USA; 2 Department of Pediatrics, University of Washington, 1959 NE Pacific St, Seattle, WA 98195 USA; 3 Department of Periodontics, School of Dentistry, University of Washington, 1959 NE Pacific St, Seattle, WA 98195 USA; 4 Center for Clinical and Translational Research, Seattle Children’s Research Institute, 1900 9th Ave, Seattle, WA 98101 USA; 5 Center for Immunity and Immunotherapies, Seattle Children’s Research Institute, 1900 9th Ave, Seattle, WA 98101 USA; 6 Department of Microbiology, University of Washington, 1959 NE Pacific St, Seattle, WA 98195 USA; 7 Department of Genome Sciences, University of Washington, 1959 NE Pacific St, Seattle, WA 98195 USA; 8 Department of Medicine, University of Washington, 1959 NE Pacific St, Seattle, WA 98195 USA

**Keywords:** Juvenile idiopathic arthritis, Oral health, Gingivitis, Microbiota

## Abstract

**Background:**

The oral microbiota has been implicated in the pathogenesis of rheumatoid arthritis through activation of mucosal immunity. This study tested for associations between oral health, microbial communities and juvenile idiopathic arthritis (JIA).

**Methods:**

A cross-sectional exploratory study of subjects aged 10–18 years with oligoarticular, extended oligoarticular and polyarticular JIA was conducted. Control groups included pediatric dental clinic patients and healthy volunteers. The primary aim was to test for an association between dental health indices and JIA; the secondary aim was to characterize the microbial profile of supragingival plaque using 16S rRNA gene sequencing.

**Results:**

The study included 85 patients with JIA, 62 dental patients and 11 healthy child controls. JIA patients overall had significantly more gingival inflammation compared to dental patients, as evidenced by bleeding on probing of the gingiva, the most specific sign of active inflammation (*p* = 0.02). Overall, however, there was a trend towards better dental hygiene in the JIA patients compared to dental patients, based on indices for plaque, decay, and periodontitis. In the JIA patients, plaque microbiota analysis revealed bacteria belonging to genera *Haemophilus* or *Kingella* elevated, and *Corynebacterium* underrepresented. In poly JIA, bacteria belonging to the *genus Porphyromonas* was overrepresented and *Prevotella* was underrepresented.

**Conclusion:**

Increased gingival inflammation in JIA was independent of general oral health, and thus cannot be attributed to poor dental hygiene secondary to disability. The variation of microbial profile in JIA patients could indicate a possible link between gingivitis and synovial inflammation.

## Background

Juvenile idiopathic arthritis (JIA) is a common rheumatologic disorder that can lead to significant disability. The cause is not known, precluding prevention or cure. A current hypothesis is that environmental triggers interact with specific human leukocyte antigen (HLA) and innate immunity genes associated with JIA [1].

Humans have co-evolved with microorganisms that play a pivotal role in immune development and homeostasis [2]. Crosstalk between the microbiome and the immune system has been implicated in the pathogenesis of adult-onset chronic inflammatory diseases, including rheumatoid arthritis (RA) [3, 4]. Altered oral microbial communities have been found in periodontitis, and periodontitis has been associated with RA [5, 6]. Both periodontitis and RA are characterized by innate immune activation [7]. Periodontal pathogens in the subgingival plaque, some also associated with RA, have been linked to gingival proinflammatory cytokines [8]. A causal relationship is suggested by control of RA by treating periodontitis [9, 10]. Adaptive immunity has also been implicated in the mechanism, given the association of P. gingivalis, a periodontal pathogen, with early RA, especially those patients with high levels of anticitrullinated protein antibodies ACPA [11].

Gene expression in JIA reflects innate and adaptive immune activation similar to that reported in periodontitis [12, 13]. However, a mechanistic link between the two diseases is not clearly established. Whereas periodontitis is an invasive process affecting deeper structures, leading to loss of connective tissue and bone, gingivitis is inflammation in response to the bacterial biofilm on teeth, and is reversible with treatment. Although periodontitis is rare in children, gingivitis, a chronic inflammatory precursor to periodontitis, occurs in up to 70% of children 6 to 11 years old, with higher rates during adolescence [14]. Severe childhood gingivitis in children is associated with a shift in oral bacteria to pathogenic gram-negative anaerobes and spirochetes [15]. Some, but not all studies reporting higher rates of gingivitis and dental decay in children with JIA could not rule out the possibility that poor dental hygiene was due to disability from arthralgias in fingers or temporomandibular joints (TMJs) [16–19]. Evidence for P. gingivalis has been found in multiple subtypes of JIA [20].

The concept that oral pathogens may trigger or perpetuate inflammatory joint disease in RA must be tested independently in JIA. The current study aimed to distinguish signs of periodontitis or gingivitis from other oral hygiene parameters, and to begin to describe the dental microbial communities associated with JIA. We hypothesized that we would find indicators of periodontitis and distinct bacterial dysbiosis in specific JIA subtypes.

## Methods

### Study population

Subjects were recruited at Seattle Children’s Hospital and the University of Washington/Seattle Children’s Hospital Center for Pediatric Dentistry. Subjects 10–18 years were included to ensure permanent dentition and no gingival erythema or bleeding secondary to tooth eruption. Exclusion criteria included infections, antibiotic or probiotic use within the prior 3 months, other autoimmune diseases, immunodeficiency, and malignancy.

Children in the JIA cohort satisfied the International League of Association for Rheumatology classification criteria for the diagnosis of oligoarticular, extended oligoarticular or polyarticular JIA [21], excluding enthesitis-related arthritis (ERA), psoriatic arthritis, undifferentiated arthritis, and systemic onset JIA. Subjects were enrolled in sequential order as they were seen in clinic, without selection by JIA subtype. Controls undergoing dental procedures for non-infectious causes (tooth removal for crowding, orthodontics or wisdom teeth removal) were recruited in the dental clinic. A second control group of healthy children was recruited from the community. The Seattle Children’s Hospital Institutional Review Board (approval #14045) reviewed all study activities. Informed parental permission, assent (for patients 10–17 years old), consent (for the one 18-year-old subject) and questionnaires were completed at the study visits. Smoking history was collected in sealed envelopes from subjects and parents separately.

### Clinical assessments

Joint exams were performed for the JIA patients by pediatric rheumatologists. JIA disease activity (active vs. inactive) was determined by the ACR provisional criteria for clinical inactive disease (CID) [22]. Oral exams on all subjects were performed by dental providers. The gingival indices (GI), plaque indices (PI) [23], community periodontal indices (CPI) [24], and bleeding on probing (BOP) [25] were assessed with a standard periodontal probe of permanent teeth, including the four first permanent molars, upper right and lower left central incisors, in order to avoid scoring for sulci associated with eruption as periodontal pockets. For GI and PI, four surfaces (buccal, lingual, mesial and distal) were scored per tooth. GI and PI were calculated by dividing the number of gingival inflammation- or plaque-containing surfaces by the total number of scored surfaces. BOP was scored as presence or absence for each tooth examined and divided by the total number of teeth examined. Part way through the study, we learned that the dental group was advised not to brush teeth the morning of the study visit, as per the clinical protocol for patients undergoing general anesthesia. The GI is responsive to acute changes, such as poor hygiene for as little as 12 h, but should not affect the BOP score. For this reason, the BOP was considered separately from the GI to dissect out the most specific sign of chronic gingival inflammation. The CPI was scored as the percentage of teeth examined. Decay missing filling teeth (DMFT) assessment was performed as a measurement of caries condition, as per the WHO Oral Health Survey Basic Methods [24]. Caries were scored by explorer or as clinically visible. Deciduous teeth, un-erupted teeth and teeth missing other than due to caries were not counted. If a tooth had both caries and a filling, it was marked as decay.

Patient/parent-reported health characteristics obtained by questionnaires included factors thought to play a role in oral health, (braces, gum bleeding, jaw pain, tooth pain, and mouth breathing). Smoking is a known risk factor for both periodontitis and rheumatoid arthritis [26, 27], and hence, patient and household cigarette smoking reports were solicited. ‘Smoke currently’ was defined as having smoked at least one cigarette in the last week or on at least one day in the last 30 days. Chronic smoking was defined as > 100 cigarettes in a lifetime.

### Oral plaque collection and DNA extraction

Plaque samples were collected by sterile endodontic paper points (Dentsply Sirona, York, PA) placed along the gingival margin of all 6 selected teeth on 22 patients with JIA (8 polyarticular, 10 oligoarticular, 4 extended oligoarticular) and 10 healthy controls. Because children have not developed periodontal pockets and thus lack accessible subgingival plaque, samples from the supragingival region were collected. Deoxyribonucleic acid (DNA) was extracted using the PowerSoil DNA Isolation Kit (MoBio, Carlsbad, CA) with a modified protocol: Prior to the addition of lysis buffer, Solution C1 (lysozyme) was added and incubated at 37 °C for 30 min, then 65 °C and 95 °C for 10 min each. For mechanical disruption, 0.5 g of 0.1 mm diameter Zirconia/Silica beads (BioSpec Products, Bartlesville, OK) were used. DNA was quantified using the Qubit 2.0 Fluorometer (Life Technologies, Carlsbad, CA).

### Library construction and 16 s rRNA sequencing

Amplicon libraries of the 16S rRNA gene were generated [28]. Briefly, the V4 region was amplified with primers containing overhang sequence compatible with Illumina sequencing technology (Illumina, Inc., San Diego, CA). The amplicons were normalized using the SequalPrep Normalization Kit (Invitrogen), pooled in equal molar amounts, quantified using the Qubit 2.0 fluorometer (Life Technologies, Carlsbad, CA) and validated with the 2200 TapeStation (Agilent Technologies, Santa Clara, CA). The final amplicon pool was diluted, mixed with the Illumina-generated PhiX control library, and denatured according to standard Illumina protocol. Sequencing was performed on the Illumina MiSeq with the addition of custom primers as described [28] generating 2 × 300 bp paired-end reads. Sequence reads were trimmed to 250 bp prior to analysis to remove the adapter sequence.

### Microbiome analysis

Of the 32 samples sequenced, sequence output data was adequate for 27. For core diversity analysis, only samples with at least 2700 sequence reads were used (random sampling rarified data to 2700 sequences each). The reads (4.14 million total) were quality filtered. Quantitative Insights Into Microbial Ecology (Qiime version 1.9.1) software was used for microbial analysis of the raw sequencing data [29]. Specific sequences that classified to the following organisms not known to be in the oral microbiota or common kit contaminants were removed: Enterobacteriaceae, Pseudomonadaceae, and Deinococcus [30]. The sequence length (151 bp) that remained after quality filtering allowed analysis of the microbiome data only to the genus level. Diversity analysis was performed based on operational taxonomic units (open references using 20 minimum sequences for an OTU and clustering using 97% similarity in OTUs). The principal coordinate analysis plot (PCoA) was created using weighted unifrac distances between samples. The linear discriminant analysis effect size with the LEfse tool was created using normalized relative abundance to show differences in enriched microbes between the two groups [31]. Only microbial communities with a two log-fold difference or greater are shown in the LEfSe plot.

### Statistical analysis

The statistical package R (version 3.3.2) was used for analysis of demographic and dental indices data. Demographic characteristics and patient reported health outcomes were compared between groups using analysis of variance (ANOVA) for quantitative variables and Fisher’s exact test for categorical variables. Linear regression analysis was used to compare dental indices across groups with adjustment for demographic characteristics (age, gender, Caucasian race, household income and highest parent education). Formal comparisons of the mean values for dental indices were made between the JIA, dental and the healthy control groups. The analysis of similarities (ANOSIM) was used to test for statistical significance from the weighted unifrac distance matrix (PCoA plots). The ANOVA model was used to analyze the differences among bacteria genera between the polyarticular JIA and healthy control groups. *P*-values < 0.05 were considered significant.

## Results

### Patient characteristics

A total of 158 sequential subjects were enrolled in the study: 85 patients with JIA, (22 oligoarticular, 17 extended oligoarticular and 46 polyarticular), and 62 dental patients. Eleven healthy controls were recruited from among the friends of JIA patients. Demographic and patient-reported health characteristics are reported in Table 1.

**Table 1:** Demographic data and patient-reported health characteristics

	Healthy Controls (*N* = 11)	Dental Cases (*N* = 62)	JIA Cases (*N* = 85)	*P*-Value
^a^Age (yrs.), mean (SD)	14.0 (2.4)	15.1 (2.3)	14.0 (2.2)	0.01
^a^Female Gender, n (%)	2 (18.2%)	39 (62.9%)	69 (81.2%)	< 0.001
^a^Caucasian race, n (%)	11 (100.0%)	33 (54.1%)	65 (78.3%)	0.03
^a^Household income >$100,000, n (%)	9 (81.8%)	6 (10.3%)	24 (30.8%)	< 0.001
^a^Parental Education >college, n (%)	8 (72.7%)	5 (8.3%)	18 (22.2%)	< 0.001
Braces, n (%)	1 (9.1%)	24 (38.7%)	20 (23.8%)	0.06
Gums bleed, n (%)	8 (72.7%)	26 (41.9%)	38 (45.2%)	0.19
Jaw pain, n (%)	2 (18.2%)	19 (30.6%)	33 (39.3%)	0.30
Tooth pain, n (%)	4 (36.4%)	22 (35.5%)	22 (26.2%)	0.41
Mouth breather, n (%)	2 (18.2%)	18 (29.0%)	29 (34.5%)	0.57
Patient smoking				
^a^Ever smoked, n (%)	0 (0.0%)	9 (14.5%)	0 (0.0%)	< 0.01
Smoke currently, n (%)	0 (0.0%)	4 (6.5%)	0 (0.0%)	0.06
^b^Chronic smoker, n (%)	0 (0.0%)	2 (3.2%)	0 (0.0%)	0.29
Household smoking				
^a^Smoke currently, n (%)	0 (0.0%)	20 (32.2%)	11 (12.9%)	< 0.01
^b^Chronic smoker, n (%)	3 (27.3%)	29 (46.8%)	30 (35.3%)	0.27

*JIA* Juvenile Idiopathic Arthritis

^a^Characteristics that were statistically different between groups (*P* < 0.05)

^b^Chronic smoking was defined as ever having smoked ≥100 cigarettes

### Oral health

Periodontitis is exceedingly rare in immunocompetent children, and we did not detect significant periodontal disease (with gingival sulcus depths > 3 mm) in any subject. We did find that JIA patients tended to have higher periodontal indices, more similar to dental patients than to healthy controls. A CPI score > 0 was present in 50.6% of JIA subjects, 55.4% of dental subjects and 36.4% of healthy controls (mean scores of 0.20, 0.28 and 0.12, respectively). The differences between groups were not significant using logistic regression and a likelihood ratio test (*p* = 0.35).

**Fig. 1:**
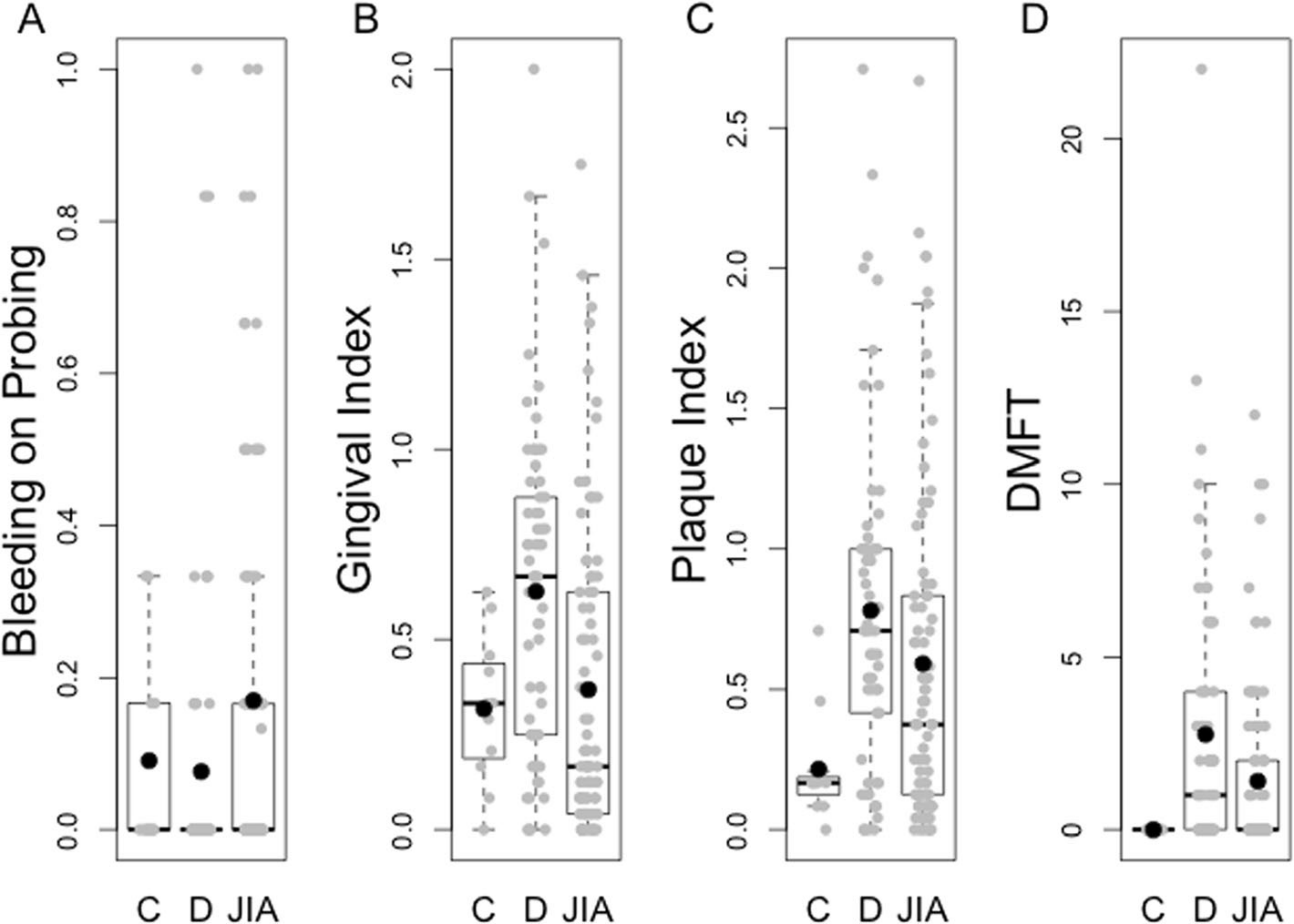
Comparison of dental indices between groups. Comparison of dental indices between the Healthy Control (**c**), Dental (**d**) and Juvenile Idiopathic Arthritis (JIA) groups in Bleeding on Probing, Gingival Index, Plaque Index, and DMFT (Decay missing filling teeth) scores. Significant differences between JIA and Dental groups were found in BOP (*p* = 0.02) and GI scores (*p* = 0.002). Black bars, medians; filled circles, means

We examined signs of gingival inflammation that may serve as early precursors to periodontitis. JIA patients had significantly higher BOP scores than dental patients (mean, (standard deviation, SD) 0.170 (0.248) vs. 0.076 (0.208)) after adjustment for demographic characteristics (*p* = 0.02, Fig. 1a), suggesting that gingivitis is increased in JIA. In a linear regression analysis (Additional file 1: Table S2), the adjusted difference between mean BOP scores in JIA patients versus dental patients was 0.104 with a standard error of 0.043 (*p* = 0.02). The JIA cohort trended toward higher mean BOP scores than the healthy control group, but this was not statistically significant (*p* = 0.41).

Additional aspects of oral health were assessed using specific dental indices (Fig. 1, Table 1, and Additional file 1: Table S1). We found that the JIA patients did not have overall worse oral hygiene, but rather better gingival indices compared to the dental group (*p* = 0.002) and non-significant trends toward better plaque and DMFT indices. Compared to healthy controls, the JIA group had significantly worse plaque indices (*p* = 0.049) and non-significant trends toward worse gingival and DMFT indices.

We investigated environmental factors that could influence outcomes. Within this study, the highest levels of education and income were noted in the healthy control group, followed by JIA and dental groups (Table 1). Covariate analysis within the dental cohort showed that the socioeconomic status (SES) influenced only the GI (0.81 in the lower income group vs. 0.37 in the higher income group, *p* <  0.05).

Only nine subjects self-reported smoking, all in the dental cohort. In addition, dental controls had significantly higher exposure to current smokers in the household (*p* <  0.01), compared to the other two groups. We found that overall, patient smoking in the dental group was associated with DMFT (*p* <  0.01), but not gingivitis, plaque, or periodontitis. In the JIA patients with household member chronic smoking, higher DMFT scores were also found (*p* <  0.01). No associations were detected between household smoking status and other dental indices.

### JIA disease activity and oral health

If gingival inflammation triggers systemic inflammation leading to synovitis, we might expect to detect a relationship between BOP and JIA disease activity. To test this hypothesis, we compared JIA patients with clinically inactive and active disease (*n* = 46, 45) [22]. No association was found between JIA disease activity and any of the dental indices measured. In addition, there were no associations between dental index scores and subtypes of JIA (oligoarticular, polyarticular, extended oligoarticular) or disease duration (average of 5.5 years). Only four JIA patients, all with polyarticular JIA, had a history of anti-citrullinated peptide antibodies (ACPA), and seven of rheumatoid factor. There was no increase in BOP in these patients compared to the other JIA patients.

We studied the effects of medications in the JIA cohort. In regression analysis we found no significant association between dental indices and either methotrexate (*n* = 38), biologics (*n* = 31), or corticosteroids (*n* = 6). Non-steroidal anti-inflammatory drugs (NSAIDs) predispose to bleeding through altered platelet function, and thus could contribute to BOP. Although 16% of the JIA patients were taking NSAIDs, we detected no increase in BOP in this group.

### Plaque microbial communities

**Fig. 2:**
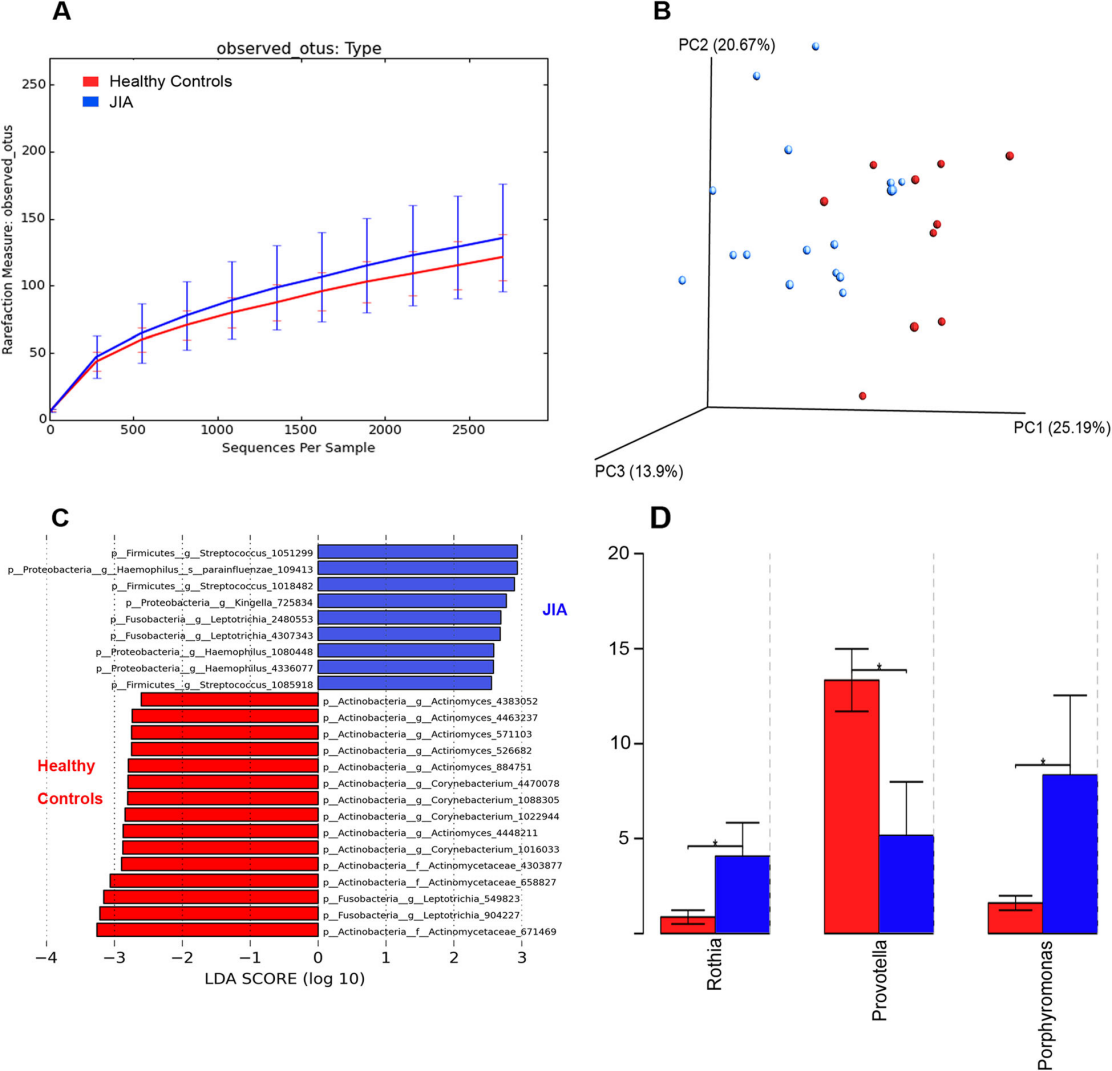
Comparison of dental plaque microbial profile between juvenile idiopathic arthritis (JIA) and healthy control groups. **a** Microbial diversity reflected by rarefraction measure of observed operational taxonomic units (OTUs) indicating trend towards higher plaque microbial diversity in JIA group (blue) compared to healthy control children (red) (*p* = 0.5); **b** Principal coordinate analysis (PCoA) showing microbial clustering of the two groups (*p* = 0.46). The first two components (Principal coordinate axis 1 and 2, PC1 and PC)) explain a total of 45.86% of the variation (first component, 25.19%, second component, 20.67%); **c** Linear discriminant analysis (LDA) through the LefSe tool. Enriched microbial communities between the two groups with a two log-fold difference or greater are shown in the LEfSe plot. **d** Bacteria genera found to be significantly different between the polyarticular JIA and healthy control groups.*p-value < 0.05

We observed a trend toward higher plaque microbial diversity (using rarefraction measure of observed OTUs) in the JIA population compared to the healthy controls (Fig. 2a). However, the alpha diversity measure between the two groups calculated using the pairwise Wilcoxon test did not show statistical significance (*p* = 0.5). Since the use of only a single measure can result in different estimates of magnitude of change and subsequently different inferences, we adopted a varied diversity measure approach as commonly used in microbiome area of study to provide a more complete picture of the community structure. Further diversity analysis was conducted with six different measures, including the Chao1 index, abundance-based coverage estimator (ACE), Shannon index, Simpson index, inverse Simpson index, and, Fisher’s alpha index. Using the Wilcoxon rank sum and pairwise Wilcoxon tests, no statistically significant differences were found.

Principal coordinate analysis showed visually evident clustering of JIA cohorts separate from healthy controls, but due to the small cohort and a few outliers, the difference was not statistically significant (Fig. 2b, ANOSIM *p* = 0.46). A LEfSe plot comparing the whole JIA cohort vs. the healthy control group showed variation in the enriched microbes that have at least a two-log fold difference in linear discriminant analysis (LDA) score (Fig. 2c). Bacteria belonging to the genera *Actinomyces* and *Corynebacterium* were more abundant in the healthy control group, whereas, *Streptococcus*, *Haemophilus, and Kingella* were more prominent in the JIA group. Polyarticular JIA patients clinically resemble adult RA patients more closely than other types of JIA. To test for microbial profiles previously reported in adult RA [32], we specifically compared the polyarticular JIA and healthy control groups. *Porphyromonas* and *Rothia* were more abundant in the polyarticular JIA group, and *Prevotella* was higher in the healthy control group (Fig. 2d, *p* < 0.05). A sub-analysis based on other JIA subtypes was also conducted. We found that the extended oligoarticular JIA group looked similar to healthy controls.

## Discussion

Oral inflammation has been implicated as a trigger for adult inflammatory diseases, especially RA. Previous reports suggest that indicators of gingival inflammation may be elevated in JIA [18, 20]. In this study, we found that JIA patients had increased BOP, a sign of gingival inflammation, despite otherwise superior oral health compared to children undergoing procedures in the dental clinic. BOP has been shown to correlate with risk for progression to periodontitis, size of inflammatory infiltrate in the gingival tissue, and levels of gingival proinflammatory mediators interleukins-1β, interleukin-8, and matrix metalloproteinases [33–35], and thus is the most specific clinical indicator of gingival inflammation. Children from families with higher income and parental education tend to have better oral health-related quality of life [36], yet BOP scores were higher in the JIA cohort independent of SES. The reasons for increased gingivitis in JIA are not clear. NSAID and tumor necrosis factor inhibitors therapy have been used to treat periodontitis and gingivitis [37–39], and thus might decrease gingival or periodontal indices in this study. On the other hand, immunosuppression could theoretically allow for expansion of microbial communities normally checked by host immunity, similar to that seen in patients with primary immunodeficiency. Previous studies have suggested that JIA patients have poor oral health because of disability preventing adequate oral hygiene [16–18]. However, BOP scores in the JIA cohort were not affected by immunosuppression, NSAID use, JIA disease activity or JIA subtype, suggesting that there is another player, perhaps the microbiota, which could contribute to the pathogenesis of both JIA and gingivitis. Microbial communities can contribute to chronic local and systemic inflammation through altered mucosal immunity or protein citrullination [5, 6].

Comparison of oral microbial communities in the pediatric population with communities previously reported in RA is difficult, because the lack of deep sulci in children prevents collection of truly subgingival plaque. Periodontal pathogens are more commonly detected in deeper pockets [40]. Moreover, the analysis of the microbime in the current study was only specific to the genus level, precluding confident identification of known periopathogen species such as *Porphyromonas gingivalis.* While we did not find a predominance of known pathogens associated with severe periodontitis [41], mainly *Prevotella intermedia*, *Fusobacterium nucleatum*, *Porphyromonas gingivalis*, *Tannerella forsythia* and *Treponema denticola* in the combined JIA cohort, we did find bacteria belonging to the *genus Porphyromonas* in the polyarticular JIA group, similar to a previous report, in which a specific assay detected *P. gingivalis* in 21.5% of JIA patients [20]*.* Bacteria belonging to genera *Haemophilus* or *Kingella*, both gram-negative microbes, were elevated in the JIA cohort, interesting because of the known shift toward gram-negative anaerobes and spirochetes in periodontitis [15]. While not statistically significant, we failed to reject the null hypothesis that microbial diversity is similar in both groups. The JIA cohort tended towards a higher microbial diversity, similar to that previously found in gingiva with periodontitis or ulcerative gingivitis [42]. In new-onset RA, subgingival *Prevotella* and *Leptotrichia* were over-represented, whereas *Corynebacterium* and *Streptococcus* were underrepresented [4]. In contrast, we found *Leptotrichia* in both JIA and control groups, and *Prevotella* was lower in the polyarticular JIA group. Similar to adult RA, *Corynebacterium* was under-represented in JIA. It is important to recognize that the pathogenic processes at play in JIA are likely different than those in RA and the associations reported in RA may only be relevant for the subset of patients with seropositive JIA. In our study, only four patients had ACPAs and seven were RF positive, all of whom had polyarticular JIA.

Similarities exist between the pathogenesis of periodontal disease and JIA. Humoral immune responses to periodontal pathogens *Porphyromonas gingivalis*, *Prevotella intermedia,* and *Aggregatibacter actinomycetemcomitans* have been found in RA and JIA, and may contribute to pathogenesis [20, 43, 44]. Moreover, abundant evidence suggests that bidirectional crosstalk exists between microbial communities and the host immune response. It is also possible, therefore, that the association between oral inflammation secondary to specific microbiota and JIA are parallel processes with a common underlying immune defect, rather than a causal relationship. To this point, while induction of periodontitis can lead to arthritis in susceptible mice [45, 46], experimental arthritis can also trigger periodontitis [47]. Defective innate immune responses could play a role in proliferation of a specific microbiota that could in turn perpetuate the inflammatory responses. It is unclear whether JIA-associated immune dysfunction allows expansion of pathogenic bacteria that lead to activation of innate immunity.

The study has limitations that affected our ability to detect more notable associations of etiopathogenic importance. This is a cross-sectional study of a diverse population of treated patients, studied a few years after diagnosis of JIA. Further, the microbiota investigation was conducted on a smaller subsample of patients and thus had low power. The findings do not pertain to all JIA subtypes, as patients with ERA, psoriatic, systemic and undifferentiated categories were excluded. Unpredicted mismatches between JIA and control groups can influence oral health and microbial flora, including SES, race, smoking, and time since last brushing. The healthy control cohort had overall SES similar to the JIA cohort, but the sample size was small. No information was collected to estimate the frequency of dental care (i.e., time since last dental examination or cleaning, diet, mouthwash use). Because smoking has been established as a contributing factor to periodontitis and RA, [26, 27] we investigated the association of smoking with dental indices, but very few subjects reported smoking, making evaluation of the impact on arthritis risk or oral hygiene impossible. The study did examine for the first time the association of second-hand smoke with gingival inflammation in JIA, and no relationship was detected. TMJ arthritis could contribute to poor oral hygiene because of difficulty in mouth opening, as suggested previously [18]. However only three patients had abnormal TMJ findings on clinical examination in the cohort reported here, and magnetic resonance imaging was not routinely conducted. Finally no corrections were made for multiple comparisons in this exploratory microbiome study, so suggested associations require confirmation in additional patient cohorts.

## Conclusions

In this study, gingival inflammation and altered dental plaque microbial communities were associated with JIA despite overall normal oral health, and thus cannot be attributed to poor dental hygiene secondary to disability. The importance of monitoring and controlling oral health in children with JIA has yet to be determined through intervention trials. Understanding the role of oral pathogens in triggering and perpetuating chronic inflammation in JIA could lead to better approaches to prevention and treatment, but will require a better understanding of the immune responses to candidate oral microbes.

## Supplementary information

**Additional file 1: Table S1**. Mean and median data for Bleeding on Probing (BOP), Gingival Index (GI), Plaque Index (PI), and Decay, Missing, Filling Teeth (DMFT) scores between the three groups. The JIA group had lower GI scores compared to the dental group (*p* = 0.002) and higher PI scores (*p* = 0.049) compared to healthy controls. No other differences were statistically significant. **Table S2.** Linear regression analysis to assess associations between oral health outcomes and patient group (JIA versus Dental), and demographic characteristics (regression coefficient estimate ± SE, *p*-value).

## Data Availability

The datasets used and/or analyzed during the current study are available from the corresponding author on reasonable request.

## Abbreviations

ACE: Abundance-based Coverage Estimator
ANOSIM: Analysis of Similarities
ANOVA: Analysis of Variance
Anti-TNF: Anti-Tumor Necrosis Factor
BOP: Bleeding on Probing
CID: Clinically Inactive Disease
CPI: Community Periodontal Index
DMFT: Decay Missing Filling Teeth
DNA: Deoxyribonucleic Acid
ERA: Enthesitis Related Arthritis
GI: Gingival Index
HLA: Human Leukocyte Antigen
JIA: Juvenile Idiopathic Arthritis
LDA: Linear Discriminant Analysis
LEfSe: Linear Discriminant Analysis Effect Size
NSAIDs: Non-steroidal Anti-Inflammatory Drugs
OTU: Operational Taxonomic Unit
PCoA: Principal Coordinate Analysis
PI: Plaque Index
RA: Rheumatoid Arthritis
SES: Socioeconomic Status
TMJ: Temporomandibular Joint
