# Supplementary material for: Oral health and plaque microbial profile in juvenile idiopathic arthritis
Source: Pediatr Rheumatol Online J. 2019 Dec 16;17:81. doi: 10.1186/s12969-019-0387-5 (PMC6916162; doi:10.1186/s12969-019-0387-5)
Supplement: Supplementary file 1 — Additional file 1: Table S1. Mean and median data for Bleeding on Probing (BOP), Gingival Index (GI), Plaque Index (PI), and Decay, Missing, Filling Teeth (DMFT) scores between the three groups. The JIA group had lower GI scores compared to the dental group (p = 0.002) and higher PI scores (p = 0.049) compared to healthy controls. No other differences were statistically significant. Table S2. Linear regression analysis to assess associations between oral health outcomes and patient group (JIA versus Dental), and demographic characteristics (regression coefficient estimate ± SE, p-value). [file 12969_2019_387_MOESM1_ESM.docx]

**Additional file 1**

**Table S1:** Mean and median data for Bleeding on Probing (BOP), Gingival Index (GI), Plaque Index (PI), and Decay, Missing, Filling Teeth (DMFT) scores between the three groups. The JIA group had lower GI scores compared to the dental group (p=0.002) and higher PI scores (p=0.049) compared to healthy controls. No other differences were statistically significant.

| **Dental Index** | **JIA (N= 85)** | | **Dental (N=62)** | | **Healthy Control (N=11)** | |
| --- | --- | --- | --- | --- | --- | --- |
|  | **Mean (SD)** | **Median** | **Mean (SD)** | **Median** | **Mean (SD)** | **Median** |
| **BOP** | 0.170 (0.248) | 0 | 0.076 (0.208) | 0 | 0.091 (0.137) | 0 |
| **GI** | 0.369 (0.407) | 0.166 | 0.627 (0.429) | 0.666 | 0.318 (0.197) | 0.333 |
| **Plaque Index** | 0.592 (0.607) | 0.375 | 0.780 (0.599) | 0.708 | 0.216 (0.199) | 0.167 |
| **DMFT** | 1.402 (2.643) | 0 | 2.758 (4.011) | 1 | 0.000 (0.000) | 0 |

**Table S2.**  Linear regression analysis to assess associations between oral health outcomes and patient group (JIA versus Dental), and demographic characteristics (regression coefficient estimate ± SE, p-value).

| **Variable** | **Bleeding on Probing** | **Gingival Index** | **Plaque Index** | **DMFT** |
| --- | --- | --- | --- | --- |
| **Intercept** | 0.20 ± 0.12, 0.10 | 0.66 ± 0.22, 0.003 | 1.46 ± 0.36, 0.000 | -1.08 ± 1.90, 0.57 |
| **Male Gender** | -0.02 ± 0.04, 0.65 | 0.06 ± 0.08, 0.47 | 0.09 ± 0.12, 0.49 | -1.31 ± 0.70, 0.06 |
| **Age** | 0.01 ± 0.01, 0.24 | 0.003 ± 0.013, 0.81 | -0.03 ± 0.02, 0.22 | 0.32 ± 0.12, 0.01 |
| **Caucasian Race** | -0.03 ± 0.05, 0.52 | 0.07 ± 0.07, 0.38 | 0.10 ± 0.11, 0.36 | -1.56 ± 0.85, 0.07 |
| **Income** |  |  |  |  |
| **< $50,000** | Reference | Reference | Reference | Reference |
| **$50,000 - $100,000** | -0.05 ± 0.04, 0.19 | -0.17 ± 0.09, 0.06 | -0.21 ± 0.12, 0.09 | 0.51 ± 0.75, 0.50 |
| **> $100,000** | -0.07 ± 0.05, 0.14 | 0.06 ± 0.11, 0.59 | -0.15 ± 0.15, 0.33 | -0.42 ± 0.86, 0.63 |
| **Parent Education** |  |  |  |  |
| **High School or Less** | Reference | Reference | Reference | Reference |
| **Vocational School or College** | -0.11 ± 0.07, 0.12 | -0.39 ± 0.11, < 0.001 | -0.51 ± 0.18, 0.004 | -0.62 ± 0.73, 0.39 |
| **Graduate or Professional** | 0.01 ± 0.09, 0.88 | -0.42 ± 0.14, 0.002 | -0.46 ± 0.20, 0.02 | -0.85 ± 1.10, 0.44 |
| **JIA Group** | 0.10 ± 0.04, 0.02 | -0.25 ± 0.08, 0.002 | -0.15 ± 0.11, 0.18 | -0.80 ± 0.67, 0.24 |
